# Synthesis and Application of Titanium Carbide (Ti3C2)-Cobalt Sulfide (Co3S4) Nanocomposites in Amino Acid Biosensing

**DOI:** 10.7759/cureus.63582

**Published:** 2024-07-01

**Authors:** Harini C P, Geetha A, Ilangovar I G K, Vasugi S, Balachandran S

**Affiliations:** 1 Department of Physiology, Saveetha Dental College and Hospitals, Saveetha Institute of Medical and Technical Sciences (SIMATS) Saveetha University, Chennai, IND

**Keywords:** x-ray diffraction, field emission scanning electron microscope, l-arginine, biosensing, titanium carbide

## Abstract

Background

The fabrication of titanium carbide (Ti_3_C_2_)-cobalt sulfide (Co_3_S_4_)-based biosensors with high sensitivity and selectivity can change the biosensor manufacturing industry completely. Molecular and clinical diagnostics, disease progression monitoring, and drug discovery could utilize these sensors for early biomarker detection. MXene (Ti_3_C_2_) is a two-dimensional material with exceptional electrical conductivity, hydrophilicity, great thermal stability, large interlayer spacing, and a high surface area. Ti_3_C_2_'s remarkable characteristics make it well-suited for biomolecule immobilization and target analyte detection. Co_3_S_4_ is a transition metal chalcogenide that has shown great potential in biosensors. Co_3_S_4_ nanoparticles (NPs) can potentially enhance Ti_3_C_2 _electrocatalytic activity, particularly in amino acid detection. L-arginine is a semi-essential amino acid, and the body frequently uses it to support healthy circulation and plays a crucial role in protein synthesis. We fabricated the Ti_3_C_2_-Co_3_S_4_ biosensor for L-arginine detection.

Aim

This study aims to synthesize and apply Ti_3_C_2_-Co_3_S_4_ nanocomposites in amino acid biosensing.

Materials and methods

The Ti_3_C_2_ nanosheets were synthesized by the selective removal of an aluminum (Al) layer from the precursor (Ti_3_AlC_2_) using hydrofluoric acid (HF). The resulting mixture serves as an etchant, especially targeting the Al layers on Ti_3_AlC_2_ while protecting the desired MXene layers at room temperature. Cobalt nitrate hexahydrate was dissolved in deionized water. Sodium hydroxide was added to the cobalt solution and stirred. Thioacetamide was added to the above solution and stirred (Solution B). A mixture of Solution A and Solution B was stirred for 30 minutes. The mixture is transferred to a hydrothermal reactor and maintained at a temperature of 180°C for 12 hours. Once the reaction completes, we cool the resultant mixture to room temperature and then filter it using the washing technique. The sample underwent a 12-hour drying process at 80°C.

Results

This study investigated the use of a biosensor that employed Ti_3_C_2_-Co_3_S_4_ NPs to detect the concentration of L-arginine. The X-ray diffraction (XRD) shows clear and distinct peaks, which means that the synthesized Ti_3_C_2_-Co_3_S_4_ nanostructures have a crystalline structure. Scanning electron microscopy (SEM) analysis revealed that the sheetlike structure of synthesized Ti_3_C_2_-Co_3_S_4_ nanostructures revealed the crystalline morphology. The results of this study show that the Ti_3_C_2_-Co_3_S_4_ NP-based biosensor can be used to detect L-arginine in a sensitive and selective way.

Conclusion

This study investigated the synthesis of Ti_3_C_2_-Co_3_S_4_ NPs and their ability to detect L-arginine levels and show a distinct correlation between the L-arginine concentration and the fluorescence intensity, demonstrating the biosensor's effectiveness in detecting L-arginine levels.

## Introduction

Amino acids are important elements of the human body that are fundamental, integral, and essential to health. Amino acids are utilized as biocompatible materials for an array of applications, including drug delivery, diagnostics, human health, nutrition, and imaging. They are also significant components of body metabolism [1,2]. L-Arginine is a semi-essential amino acid crucial for regulating various metabolic processes and boasts a diverse range of biological functions. It serves as a precursor to several important compounds, such as L-ornithine, γ-amino butyric acid, and spermine. Notably, L-arginine is one of the most highly charged positively polarized amino acids. This amino acid is vital for our metabolic processes and plastic metabolism. It is also responsible for the synthesis of nitric oxide, certain hormones, polyamines, and creatine. Additionally, it acts as a precursor for the formation of other amino acids such as proline, glutamate, and glutamine. L-Arginine is capable of endogenous synthesis in the body; however, its synthesis decreases considerably with age-related changes and in some pathological conditions [3,4].

L-arginine is a chemical that has great importance in clinical and quality control applications. It is mostly located at the active sites of many proteins. The structure of the enzyme is conducive to facilitating the attachment of phosphate anions, which in turn enables the catalysis of phosphorylation processes. Additionally, arginine is crucial in regulating the electrical charge of several proteins. Arginine is degraded into urea and ornithine during nitrogen metabolism by the activity of arginase. Arginine also assists in the process of removing ammonia from the body as well as in the release of hormones and the maintenance of the immune system. According to reports, the process of converting arginine into nitric oxide through nitric oxide synthase can potentially aid in the treatment of various physiological conditions including cardiovascular diseases, peripheral vascular disease, erectile dysfunction, atherosclerosis, vascular headaches, and chest pain. This is achieved by improving vasodilation, or the widening of blood vessels. Arginine stimulates protein synthesis, prevents tissue degradation, and promotes spermatogenesis [5,6].

L-Arginine has a diverse array of effects on the functioning of different systems, making it a potential therapy choice for a range of disorders. These disorders include illnesses related to cardiology, diseases of the central nervous system and liver, and even respiratory disorders. There has been an increase in the occurrence of counterfeit medications, despite the availability of high-quality treatments. These counterfeit medicines typically contain incorrect proportions of active chemicals or may contain no active compounds at all. Hence, regulating the content of arginine in medicines is of the utmost importance. It is necessary to measure the concentration of L-arginine in biological fluids. To determine the concentration of L-arginine in solutions, various methods, including spectrophotometry, ion-exchange chromatography, and high-performance liquid chromatography (HPLC), are used. Most of the existing physicochemical and chemical techniques have drawbacks, including limited selectivity and sensitivity, as well as the need for expensive and complex equipment. To address these issues and reduce costs, biosensors represent a highly promising alternative [7].

An important demand for the field of biosensors is the development of sophisticated sensors that possess exceptional sensitivity and selectivity. Several nanomaterials have been developed to build biosensors that possess exceptional sensitivity and selectivity, fulfilling the required parameters. MXene (Ti_3_C_2_) is a highly regarded nanomaterial that is garnering attention for its excellent characteristics, resulting in a perfect choice for designing biosensors. Ti_3_C_2_ is a two-dimensional (2D) material, is a transition metal carbon, nitride and carbonitrides. It has gained significant interest in various fields, including supercapacitors, Li-ion batteries, catalysts, transparent conductors, biomaterials, lubricants, field-effect transistors, sensors, drug carriers, dual-responsive surfaces, EMI shielding materials, purifiers, hybrid nanocomposites, dye substrates, and cancer treatment [8]. This is because Ti_3_C_2_ possesses distinct properties such as hydrophilicity, biocompatibility, conductivity, and stability. Ti_3_C_2 _are produced by selectively etching the A layer from the Ti_3_AlC_2_ MAX phase using a chemical etchant. In this process, M represents the transition metal materials, A represents the elements from the III A and Ⅵ A columns, and X represents carbon and/or nitrogen elements. The layers consist of C atoms arranged between the octahedral sites, and Ti_n+1_C_n_ layers are alternating with layers of Al atoms. The Ti-C bond exhibits a strong bonding of covalent/metallic/ionic character, while the Ti-Al layers are weakly bonded and contain a pure metallic nature. As a result, the Ti-Al bond has a tendency to break down at elevated temperatures, forming Ti_n+1_C_n_ compounds. This process promotes recrystallization and the creation of a three-dimensional (3D) rocksalt-like structure of Ti_n+1_C_n_. Ti_3_C_2_ is the most extensively researched MXene because of its ease of synthesis and high stability. Ti_3_C_2_ have been used in the development of various advanced biosensors, including electrochemical, fluorescent/optical, and surface-enhanced Raman spectroscopy (SERS) biosensors. Ti_3_C_2_ biosensor utilizes the distinctive electrocatalytic properties of the Ti_3_C_2_ sheets in response to the concentration of the target signals. The electrical properties and current signal are modified by biological targets that bind to the Ti_3_C_2_ sheets. The 2D layer nanostructure has a substantial surface area capable of accommodating biological components. The surface of the Ti_3_C_2_ nanocomposite contains active functional groups that immobilize biological components, hence altering the electrocatalytic properties and resulting in a linear response. This has been achieved by the alteration of Ti_3_C_2_ characteristics to optimize them for particular types of biosensors or by integrating them with other nanomaterials [9-12].

Cobalt sulfide (Co_3_S_4_) is an important class of transition metal chalcogenides that have many uses in many energy storage and generating technologies, including batteries, supercapacitors, and photovoltaic solar cells, as well as enzyme-free glucose sensors. The distinctive arranged morphological properties of 2D Co_3_S_4_ not only enhance the penetration of electrolyte but also offer a more reactive surface when assessed as an electrode material for biosensors. Additionally, it enables the spontaneous arrangement of thiolated compounds on its surfaces, which presents a benefit in effortless surface modification. Nevertheless, Co_3_S_4_ is unsuitable for usage in electrode materials due to its semiconductor nature and low conductivity. Therefore, it is extremely advantageous to create a composite of Co_3_S_4_ with materials that are electrically conductive in order to enhance the transfer of charges for applications in electrochemical biosensing [13]. Co_3_S_4 _has excellent biocompatibility, high electrocatalytic activity, and significant adsorption capacity, making it highly desirable for sensing fabrications. Despite its potential, Co_3_S_4_ is hindered by its low electrical conductivity, which restricts its capacity to exhibit high sensitivity, reliability, and rapid response times [14].

A proposed method to combat these issues is to mold the Co_3_S_4_ into a Ti_3_C_2_, which would highlight the advantageous characteristics of both materials and reduce the drawbacks they have. The synergistic effect of the two existing materials will eliminate the problem of low sensitivity in Ti_3_C_2_ and the poor selectivity in Co_3_S_4_. Co_3_S_4_ nanoparticles (NPs) have the ability to facilitate redox reactions that are crucial in electrochemical biosensing. On the other hand, Ti_3_C_2_ can improve the catalytic efficiency as a result of their large surface area and conductivity. The combination of these materials may result in enhanced catalytic activity and accelerated response times in biosensors, due to their synergistic impact. Ti_3_C_2_-Co_3_S_4_ improve the overall conductivity of the sensor. Enhancing the efficiency of electron transfer processes in biomolecular contacts can result in improved sensitivity and biosensing applications. This study focuses on the synthesis of a Ti_3_C_2_-Co_3_S_4_, which involves combining Ti_3_C_2_ and Co_3_S_4_ materials. Confocal fluorescence microscopy is used to further investigate possible uses of this nanomaterial in L-arginine sensing.

## Materials and methods

Materials

The study materials included the following: titanium aluminum carbide (Ti_3_AlC_2_), hydrofluoric acid (HF), deionized water, ethanol, acetone, cobalt (II) nitrate hexahydrate (Co(NO_3_)_2_·6H_2_O), sodium hydroxide (NaOH), thioacetamide (CH_3_CSNH_2_), Teflon-lined stainless steel autoclave.

Synthesis of Ti_3_C_2_ MXene

Ti_3_C_2_ MXene was synthesized using the Ti_3_AlC_2_ MAX phase, which was obtained commercially, as the initial material. The 2.5 g of Ti_3_AlC_2 _black powder was immersed in 60 mL of 40% HF for 24 h. A magnetic stirring process was used to maintain heating (40°C) and stirring throughout the whole etching process, with 24 h of etching time and increased reaction efficiency. An amount of about 10 cm^3^ of HF per 1 g of the initial material was used. Small amounts of Ti_3_AlC_2_ were introduced into HF during the reaction. A highly exothermic reaction and significant hydrogen evolution were observed. The resultant mixture functions as an etchant, specifically targeting the aluminum layers on Ti_3_AlC_2 _while protecting the desirable MXene layers. To neutralize the compound's acidity, the material was thoroughly washed until its pH level reached 8 (Solution A). 

Synthesis of Ti_3_C_2_-Co_3_S_4_

A total of 20 mmol of Co(NO_3_)_2_·6H_2_O were dissolved in 50 mL of deionized water in a separate beaker, and 6 mmol of NaOH were dissolved in 50 mL of deionized water in a separate beaker. NaOH solution was slowly introduced into Co solution and stirred slowly for 30 min. After the suspension was created, about 4 mmol of thioacetamide was dissolved in the solution while vigorously stirring for 30 min (Solution B). Solution B was gradually introduced into Solution A, which contained Ti_3_C_2_ MXene. A mixture of Solution A and Solution B was stirred for 30 min. The mixture is transferred to a hydrothermal reactor and maintained at a temperature of 180°C for 12 h. Once the reaction is complete, we cool the resultant mixture to room temperature and then filter it using the washing technique. The sample underwent a 12 h drying process at 80°C.

Biosensing of L-arginine

Prepare a solution by combining the 7-amino-4-methylcoumarin (AMC)-coated Ti_3_C_2_ and Co_3_S_4_ to form a fluorescent probe. To get a measurable fluorescence signal and prevent quenching effects, it is important to adjust the concentration of the probe. Create a calibration curve to establish a correlation between the intensity of fluorescence and the concentration of L-arginine. This entails creating a sequence of standardized solutions with predetermined L-arginine concentrations and quantifying their fluorescence intensity. Quantify the brightness of your samples by using a fluorescence spectrometer to measure the intensity of fluorescence. The customary procedure is to stimulate the fluorescent probe with light of the same wavelength as its excitation. Quantify the fluorescence emitted at the specific emission wavelength. Examine the fluorescence data by contrasting the fluorescence intensity of your sample with the calibration curve to ascertain the concentration of L-arginine. Create L-arginine standard solutions with different concentrations, such as 0.1, 0.25, 0.50, 0.75, and 1 mg/mL. Introduce a predetermined quantity of the fluorescent probe solution into each standard solution. Quantify the level of fluorescence intensity for each standard solution. Probes based on coumarin are highly effective. Coumarin derivatives are well-known for their remarkable sensitivity and selectivity. These compounds can be modified to show fluorescence changes in the presence of specific amino acids, like L-arginine.

## Results

X-ray diffraction (XRD) 

**Figure 1:**
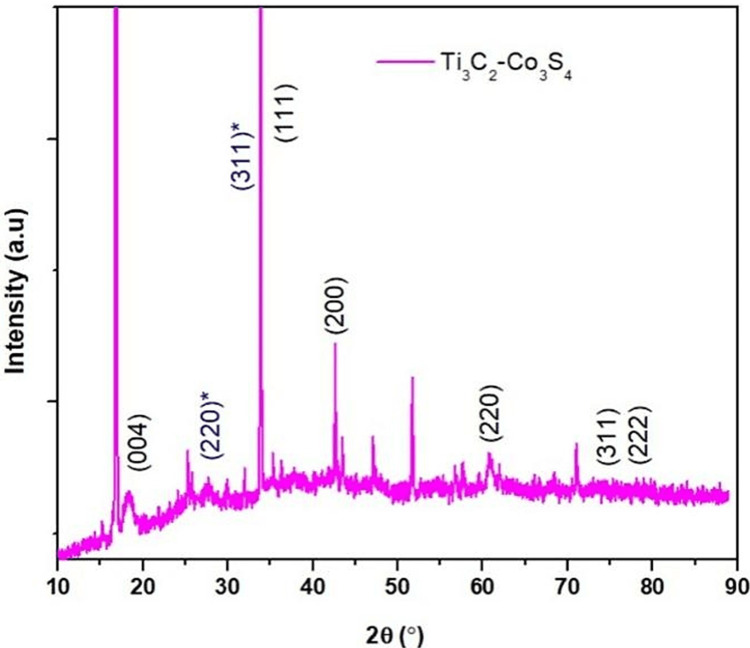
XRD analysis of Ti3C2-Co3S4 XRD: X-ray diffraction; Ti_3_C_2_-Co_3_S_4_: titanium carbide-cobalt sulfide; Θ: theta

XRD is a rapid analytical technique used to determine the crystal structure of materials. The XRD spectra of Ti_3_C_2_-Co_3_S_4 _were determined by measuring the range of 2θ from 10° to 90°. Figure 1 displays the XRD pattern of Ti_3_C_2_-Co_3_S_4_. The Co_3_S_4_ exhibits prominent diffraction peaks at 26.6° and 31.3°, which are assigned to the (220) and (311) lattice planes of the standard (JCPDS No. 42-1448). This confirms the existence of single-phase hexagonal Co_3_S_4_. The distinct and well-defined peaks indicate that the synthesized Co_3_S_4_ exhibits a good crystalline structure. The XRD analysis reveals that the peaks of Ti_3_C_2_ align with the crystal plane of (004) at an angle of 18.4° (JCPDS file No. 52-0875). Simultaneously, the distinct peak associated with the (104) lattice planes of Ti_3_AlC_2_ disappears, indicating the successful elimination of the interlayer of Al atoms in Ti_3_AlC_2_ and the transformation of Ti_3_AlC_2_ into Ti_3_C_2_. 

Field emission scanning electron microscopy (FESEM)

**Figure 2:**
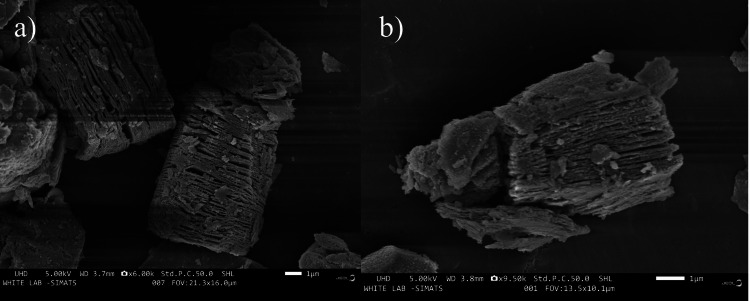
FESEM analysis of Ti3C2-Co3S4 FESEM: Field emission scanning electron microscopy; Ti_3_C_2_-Co_3_S_4_: titanium carbide-cobalt sulfide

FESEM images of Ti_3_C_2_-Co_3_S_4_ were examined using an ultrahigh-definition (UHD) microscope operating at 5.00 kilovolts (kV) with a working distance (WD) of 3.7 millimeters (mm) at 1µm. The morphology of the sample has been studied using FESEM. Figure 2a shows a sheetlike structure of HF-etched Ti_3_C_2_ MXene that is well-stacked and delaminated. The FESEM images of Ti_3_C_2_ MXene were obtained to observe the well-organized, layered structure of the nanosheets. The nanosheets are very thin and have many layers with clear, sharp edges, as shown in Figure 2b. This proves that the Ti_3_C_2_ MXene that was synthesized is of high quality. These two images clearly demonstrate that the small spherical particles observed on the Ti_3_C_2_ MXene surfaces are Co_3_S_4_. The high specific surface area of the Ti_3_C_2_ MXene allows for the adsorption of Co_3_S_4_ NPs, resulting in a homogeneous distribution of Co_3_S_4_ on the Ti_3_C_2_ MXene. The FESEM image revealed that the synthesized Ti_3_C_2_-Co_3_S_4_ has a crystalline morphology. 

Energy dispersive X-ray spectroscopy analysis (EDS) OF Ti_3_C_2_-Co3S_4_

**Figure 3:**
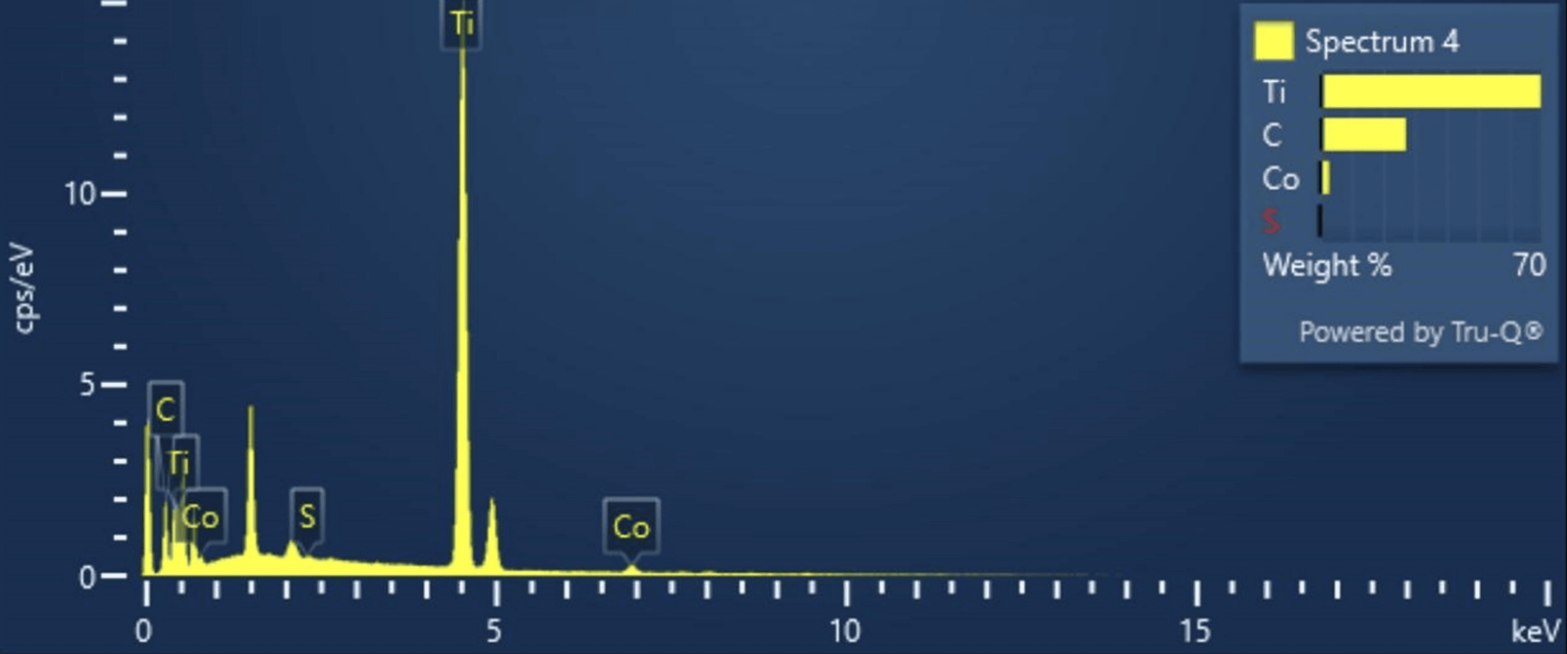
EDS analysis of Ti3C2-Co3S4 EDS: Energy dispersive X-ray spectroscopy; Ti_3_C_2_-Co_3_S_4_: titanium carbide-cobalt sulfide; Ti: titanium; C: carbon; Co: cobalt; S: sulfur

EDX, referred to as EDS or EDAX, is an x-ray method used to detect and analyze the elements in the Ti_3_C_2_-Co_3_S_4_ sample. Ti_3_C_2_ is primarily made up of titanium (Ti) and carbon (C). EDS spectrum exhibits distinct X-ray peaks that correspond to the elements present. The intensities of these peaks may be utilized to determine the relative abundance of each element. The EDS mapping results (Figure 3) show a uniform dispersion of four elements Ti, C, Co, and sulfur (S) in the Ti_3_C_2_-Co_3_S_4_ compound. The weight percentages of Ti, C, Co, and S were 69.85%, 27.19%, 2.84%, and 0.12%, respectively. The total weight of Ti_3_C_2_-Co_3_S_4_, being 100%, demonstrates its purity and the absence of impurities in the synthesized compound. EDS analysis confirms that no additional materials are present, indicating that the Ti_3_C_2_-Co_3_S_4_ is impurity-free (Table 1). 

**Table 1:** EDS spectrum analysis of Ti3C2-Co3S4 EDS: Energy dispersive X-ray spectroscopy; Ti_3_C_2_-Co_3_S_4_: titanium carbide-cobalt sulfide; Ti: titanium; C: carbon; Co: cobalt; S: sulfur; FeS2: iron(II) disulfide

Element	Line type	Apparent concentration	k ratio	Wt%	Wt% sigma	Standard label	Factory standard
C	K series	0.42	0.00418	27.19	0.47	Vit. C	Yes
S	K series	0.01	0.00007	0.12	0.05	FeS_2_	Yes
Ti	K series	4.44	0.04441	69.85	0.47	Ti	Yes
Co	K series	0.17	0.00168	2.84	0.17	Co	Yes
Total:				100.00			

Biosensing of L-arginine

**Figure 4:**
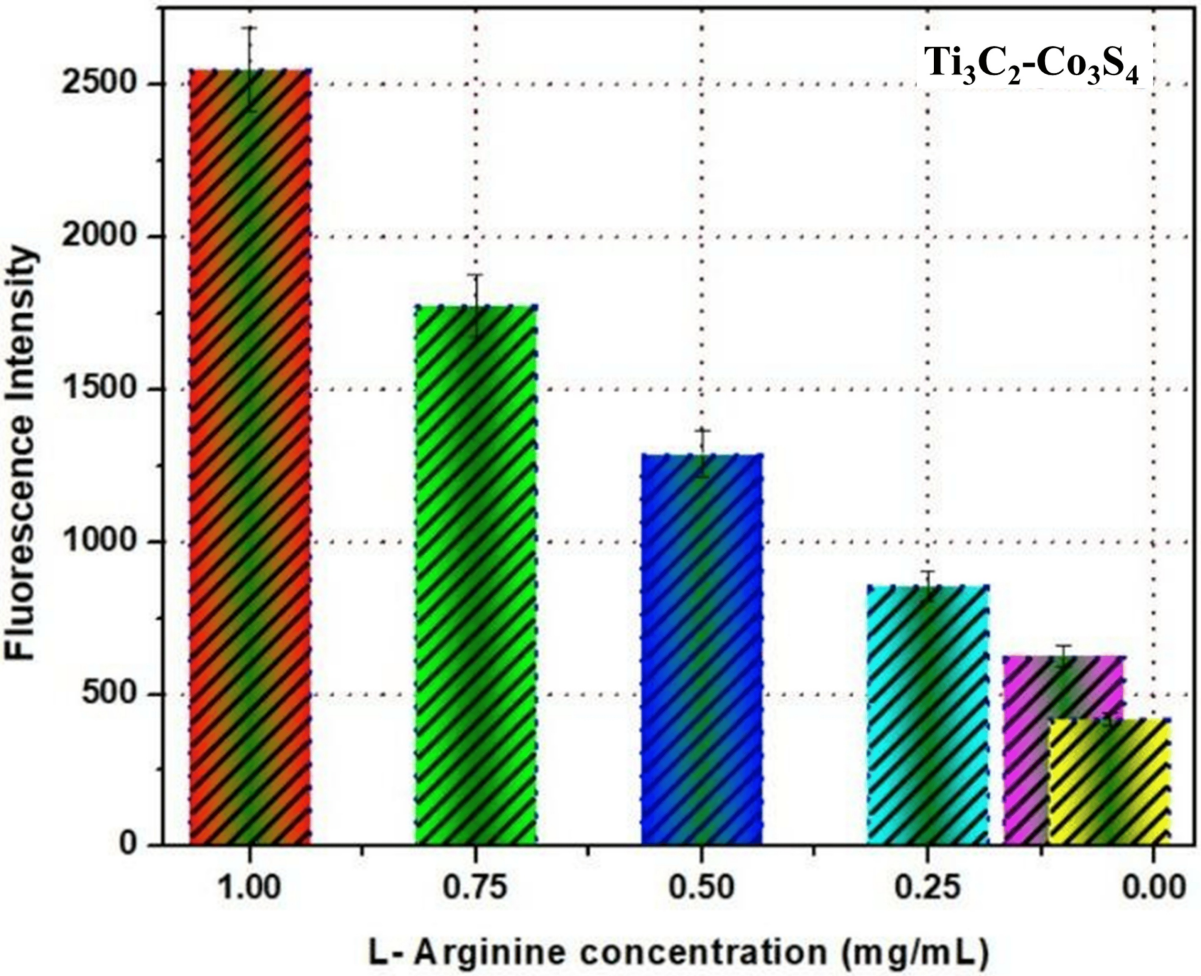
Detection of L-arginine using Ti3C2-Co3S4 biosensor with different concentrations Ti_3_C_2_-Co_3_S_4_: Titanium carbide-cobalt sulfide

Developing arginine-based fluorescent biosensors entails converting arginine into ammonia via a two-step process. The protonation of the pH-sensitive indicator (rhodamine 6G) occurs during ammonium ion production; deprotonation alters the indicator's fluorescence spectra. Confocal fluorescence microscopy is an effective imaging technique used for the biosensing of biomolecules. The technique has several benefits for biosensing, including high spatial resolution, the capability to optically segment samples, and the ability to observe dynamic processes in real time. The graph illustrates the correlation between the concentration of L-arginine and the intensity of fluorescence in a biosensor that contains Ti_3_C_2_-Co_3_S_4_ NPs. It was examined using confocal fluorescence microscopy. The Ti_3_C_2_-Co_3_S_4_ biosensor, as seen in Figure 4, is capable of detecting L-arginine even at low concentrations. At a concentration of 0.50 mg/mL, the fluorescence intensity continues to increase compared to 0.25 mg/mL. At a concentration of 1.00 mg/mL, the fluorescence intensity increases further compared to 0.75 mg/mL. As the concentration of L-arginine increases, there is a noticeable increase in fluorescence intensity. The results demonstrate a concentration-dependent correlation between L-arginine concentration and fluorescence intensity in the L-arginine biosensor utilizing Ti_3_C_2_-Co_3_S_4_ NPs. An L-arginine-specific fluorescent probe is functionalized into the Ti_3_C_2_-Co_3_S_4_ heterostructure. Adsorption or covalent attachment of a fluorophore that changes its fluorescence intensity upon binding to L-arginine may accomplish this. Common fluorophores include rhodamine. A glass substrate that is appropriate for confocal fluorescence microscopy is used to immobilize the functionalized Ti_3_C_2_-Co_3_S_4_ heterostructure. One way to do this is to pour the heterostructure dispersion onto the substrate using a dropcasting technique and then let it cure. The fluorescence characteristics of the connected probe are altered when L-arginine interacts with the Ti_3_C_2_-Co_3_S_4_ heterostructure. Fluorescence amplification was seen upon L-arginine binding to several probes, which varied in nature and interaction mechanism. L-arginine attaches to the fluorescent probe-functionalized Ti_3_C_2_-Co_3_S_4_ heterostructure at specified places. Various binding mechanisms, such as electrostatic attraction, hydrogen bonding, or covalent binding, might promote this interaction and cause the fluorescent probe to undergo a conformational shift. Confocal fluorescence microscopy is used to examine the biosensor's fluorescence response. After positioning the substrate on the microscope stage, the fluorescent probe is excited at its excitation wavelength using a laser. In order to capture high-resolution fluorescence pictures, the emission is detected using a confocal pinhole. It is possible to determine the concentration of L-arginine in a sample by measuring the intensity of its fluorescence. To determine the amount of an unknown material, calibration curves are made using known L-arginine concentrations. A sensitive and quantitative detection approach is provided by plotting the change in fluorescence intensity (quenching or amplification) against the L-arginine concentration. The Ti_3_C_2_-Co_3_S_4_ heterostructure guarantees great sensitivity and selectivity by providing a large surface area for probe attachment and an effective interaction with L-arginine.

## Discussion

Ti_3_C_2_ MXene has been recognized as an extremely advanced biosensing platform due to its high metallic conductivity, efficient ion-transmission capabilities, good biocompatibility, large surface area, and simple functionalization [15]. Xu et al. begin employing 2D materials in the field of biosensors to investigate neurological activity. They employed ultrathin Ti_3_C_2_ MXene micropatterns to create a very sensitive field-effect transistor (FET) biosensor. This biosensor can detect tiny compounds in normal biological settings without labels. Additionally, it can quickly detect action potentials in primary neurons. It presents a novel opportunity for utilizing Ti_3_C_2_ MXene in biosensing applications, highlighting its potential as a viable option for developing biosensors [16]. Chia et al. described the synthesis of a Ti_3_C_2_ MXene using HF etching and subsequent delamination with tetrabutylammonium hydroxide (TBAOH). They utilized Ti_3_C_2_ MXene as a transducer platform to create an electrochemical glucose biosensor with chronoamperometric detection. With a linear range, the biosensor demonstrated excellent selectivity and efficient electrocatalytic performance for glucose detection [17]. Wu et al. investigated the use of Ti_3_C_2_ MXene as a novel matrix for immobilizing tyrosinase, a model enzyme. This was achieved by creating a mediator-free biosensor capable of detecting phenol with high sensitivity and speed. The results have demonstrated that a surface-controlled electrochemical mechanism may readily accomplish the direct transfer of electrons between tyrosinase and the electrode. The tyrosinase biosensor that has been produced demonstrates excellent sensitivity, reproducibility, and stability. It also has a low detection limit and a large linear range. The suggested biosensing technique exhibited excellent repeatability, reproducibility, long-term stability, and high recovery when detecting phenol in actual water samples. The exceptional performances of Ti_3_C_2_ MXene, which possesses a graphene-like structure, demonstrate its durability and versatility as an electrochemical biosensing platform for enzyme-based biosensors and biocatalysis. This material has significant promise for many applications in biomedical detection and environmental analysis [18].

An enzymatic glucose detection biosensor platform utilizing a nanocomposite of gold (Au)/MXene is reported by Rakhi et al., which exhibits high sensitivity. The biosensor utilizes distinctive electrocatalytic characteristics and synergistic interactions between AuNPs and Ti_3_C_2_ MXene sheets. A glucose biosensor is produced by immobilizing the glucose oxidase (GOx) enzyme on a Nafion-solubilized Au/Ti_3_C_2_ MXene nanocomposite on a glassy carbon electrode (GCE). The biomediated AuNPs serve a crucial role in enhancing the electron transfer between the electroactive core of GOx and the electrode. The biosensor electrode consisting of GOx/Au/Ti_3_C_2_ MXene/Nafion/GCE exhibited a linear amperometric response while measuring glucose concentrations. Furthermore, the biosensor demonstrated exceptional stability, reproducibility, and repetition. Hence, the Au/ Ti_3_C_2_ MXene nanocomposite discussed in that study has promise as an electrochemical transducer for use in electrochemical biosensors [19]. Wang et al. described a biosensor that does not require a mediator to detect H_2_O_2_. The biosensor works by immobilizing hemoglobin (Hb) on an electrode that has been treated with Ti_3_C_2_ MXene. A nanocomposite of TiO_2_-Ti_3_C_2_, where TiO_2_ NPs are transformed into an organ like Ti_3_C_2_ MXene, was produced. TiO_2_-Ti_3_C_2_ nanocomposite was then utilized to trap Hb in order to create a biosensor without the need for a mediator. The spectroscopic and electrochemical data indicate that the TiO_2_-Ti_3_C_2_ nanocomposite serves as a very effective immobilization matrix for redox proteins, exhibiting outstanding biocompatibility and promoting both protein bioactivity and stability. The TiO_2_-Ti_3_C_2_ hybrid structure, which resembles an organ, promotes the direct transfer of electrons from Hb. The biosensors that were made exhibited excellent performance in detecting H_2_O_2_, with a broad linear range for H_2_O_2_ detection. Specifically, a large number of TiO_2_ NPs with exceptional biocompatibility coat the nanocomposite surface. This coating creates a protective milieu for Hb, resulting in improved long-term biosensor stability. The TiO_2_-Ti_3_C_2_ nanocomposite has great potential as a matrix for creating biosensors without the need for a mediator. It might have a wide range of applications in environmental monitoring and biological detection [20].

Additional enhancements have been accomplished by the application of metal sulfide NPs to the surface of Ti_3_C_2_ MXenes. This process effectively augments the surface area and conductivity of the electrodes while optimizing the enzyme loading. 2D transition metal chalcogenides, including copper sulfide, Co_3_S_4_, tungsten sulfide, and molybdenum sulfide, have garnered considerable interest due to their numerous benefits for basic and technological study in several disciplines, such as energy storage, sensing, and catalysis [21,22]. They consist of alternating layers of metal and S that are held together by weak van der Waals' forces. This type of material is anticipated to work exceptionally well due to the 2D electron-electron correlations between metal atoms, which can enhance planar electric conductivity [23].

The CoS/AuNPs/GCE exhibited a low baseline current, excellent conductivity, and a substantial electroactive surface area. A novel electrochemical aptasensor was subsequently developed for the detection of 17β-estradiol. The aptasensor utilized methylene blue (MB) as an indicator and cDNA, which has a high concentration of guanine, as a signal amplifier. The cDNA exhibited sequence complementarity to the 17β-estradiol aptamer, suggesting that it might potentially compete with 17β-estradiol for binding to the immobilized aptamer on the electrode surface. When 17β-estradiol forms a hybrid with an aptamer, it will reduce the extent to which cDNA binds to the aptamer, hence lowering the signal produced by MB attached to the cDNA. Conversely, the hybridization of cDNA will improve the specificity of the aptasensor [13].

The distinctive layered morphology of 2D Co_3_S_4_ enables easy penetration of electrolyte and enhances its surface activity as an electrode material for biosensors. Additionally, it allows for the self-assembly of thiolated compounds on its surfaces, providing an advantage in simple surface modification. Co_3_S_4_ is inefficient for usage in electrode materials due to its semiconductor nature and very poor conductivity. Therefore, it is extremely advantageous to produce a composite of Co_3_S_4_ with materials that have the ability to conduct electricity in order to enhance the transfer of charges for the purpose of electrochemical biosensing applications [24]. Ti_3_C_2_ MXene are highly recommended due to their ability to significantly enhance the current response of electrochemical sensors. This is attributed to their excellent conductivity and the ability to immobilize biomolecules. Fabrication of the Ti_3_C_2_-Co_3_S_4_ would not only significantly enhance its conductivity but also establish a sensing platform with amplified signal for target molecules [25]. When Co_3_S_4_ is used alone, it can act as a semiconductor and have a poor conductivity. However, when it is combined with MXene, these limitations can be reduced. The resulting material has improved capabilities for transferring charges and increased sensitivity to specific substances being analyzed. The synthesized Ti_3_C_2_-Co_3_S_4_ demonstrated favorable performance attributes, such as enhanced sensitivity, exceptional selectivity, and low detection thresholds, rendering them appropriate for amino acid biosensing [26].

Limitation

The practical implementation of these biosensors is hindered by several hurdles, including the repeatability of their results and the feasibility of mass production. The lack of published research on the in vitro and in vivo cytocompatibility of MXene-based materials poses a potential risk to their medical applications. Conventional methods of analysis only capture a single time point in samples, making it difficult to achieve real-time biosensing in vivo or intracellular using S-containing NPs.

## Conclusions

This study investigated the synthesis of Ti_3_C_2_-Co_3_S_4_ NPs and their detection of amino acids. It revealed that these NPs are efficient biosensors for detecting levels of L-arginine. XRD analysis shows clear and well-defined peaks, which show that the synthesized Ti_3_C_2_-Co_3_S_4_ nanostructures have a crystalline structure. This crystalline structure is crucial for the biosensor's stability and performance. A FESEM study reveals that synthesized Ti_3_C_2_-Co_3_S_4_ has a sheetlike structure with a highly organized arrangement. EDS analysis indicates the existence of elements such as Co, Ti, S, and C. The results show a clear relationship between the concentration of L-arginine and the fluorescence intensity, confirming the biosensor's efficiency in detecting L-arginine levels. This study illustrates the feasibility and efficiency of the Ti_3_C_2_-Co_3_S_4_ NP-based biosensor for sensitive and selective detection of L-arginine.

## References

[1] Baghal Behyar M, Hasanzadeh M, Seidi F, Shadjou N (2023). Sensing of amino acids: critical role of nanomaterials for the efficient biomedical analysis. Microchem J.

[2] Wu G (2009). Amino acids: metabolism, functions, and nutrition. Amino Acids.

[3] Marini JC (2012). Arginine and ornithine are the main precursors for citrulline synthesis in mice. J Nutr.

[4] Böger RH, Bode-Böger SM (2001). The clinical pharmacology of L-arginine. Annu Rev Pharmacol Toxicol.

[5] Verma N, Singh AK, Kaur P (2015). Biosensor based on ion selective electrode for detection of L-arginine in fruit juices. J Anal Chem.

[6] Wu G, Bazer FW, Davis TA (2009). Arginine metabolism and nutrition in growth, health and disease. Amino Acids.

[7] Soldatkina OV, Soldatkin OO, Velychko TP, Prilipko VO, Kuibida MA, Dzyadevych SV (2018). Conductometric biosensor for arginine determination in pharmaceutics. Bioelectrochemistry.

[8] Prakash NJ, Kandasubramanian B (2021). Nanocomposites of MXene for industrial applications. J Alloys Compd.

[9] Kumar S, Lei Y, Alshareef NH, Quevedo-Lopez MA, Salama KN (2018). Biofunctionalized two-dimensional Ti(3)C(2) MXenes for ultrasensitive detection of cancer biomarker. Biosens Bioelectron.

[10] Sinha A, Dhanjai Dhanjai, Zhao H (2018). MXene: an emerging material for sensing and biosensing. Trends Analyt Chem.

[11] Liu R, Jiang L, Yu Z (2021). MXene (Ti3C2T)-Ag nanocomplex as efficient and quantitative SERS biosensor platform by in-situ PDDA electrostatic self-assembly synthesis strategy. Sens Actuators B Chem.

[12] Xu B, Zhi C, Shi P (2020). Latest advances in MXene biosensors. JPhys Mater.

[13] Huang KJ, Liu YJ, Zhang JZ, Cao JT, Liu YM (2015). Aptamer/Au nanoparticles/cobalt sulfide nanosheets biosensor for 17β-estradiol detection using a guanine-rich complementary DNA sequence for signal amplification. Biosens Bioelectron.

[14] Sridhar V, Park H (2018). Carbon encapsulated cobalt sulfide nano-particles anchored on reduced graphene oxide as high capacity anodes for sodium-ion batteries and glucose sensor. J Alloys Compd.

[15] Mohammad Khazaei, Masao Arai, Taizo Sasaki (2013). Novel electronic and magnetic properties of two-dimensional transition metal carbides and nitrides. Adv Funct Mater.

[16] Xu B, Zhu M, Zhang W (2016). Ultrathin MXene-micropattern-based field-effect transistor for probing neural activity. Adv Mater.

[17] Chia HL, Mayorga-Martinez CC, Antonatos N, Sofer Z, Gonzalez-Julian JJ, Webster RD, Pumera M (2020). MXene titanium carbide-based biosensor: strong dependence of exfoliation method on performance. Anal Chem.

[18] Wu L, Lu X, Dhanjai Dhanjai (2018). 2D transition metal carbide MXene as a robust biosensing platform for enzyme immobilization and ultrasensitive detection of phenol. Biosens Bioelectron.

[19] Rakhi RB, Nayak P, Xia C, Alshareef HN (2016). Novel amperometric glucose biosensor based on MXene nanocomposite. Sci Rep.

[20] Wang F, Yang C, Duan M, Tang Y, Zhu J (2015). TiO2 nanoparticle modified organ-like Ti3C2 MXene nanocomposite encapsulating hemoglobin for a mediator-free biosensor with excellent performances. Biosens Bioelectron.

[21] Ramesh S, Ahmed ATA, Haldorai Y, Kakani V, Bathula C, Kim HS (2023). Cobalt sulfide@cobalt-metal organic frame works materials for energy storage and electrochemical glucose detection sensor application. J Alloys Compd.

[22] Li Z, Li G, Wu Z, Jiao S, Hu Z (2019). Cobalt sulfides/carbon nanohybrids: a novel biocatalyst for nonenzymatic glucose biofuel cells and biosensors. RSC Adv.

[23] Zhou Y, Xu Y, Zhang C, Emmer Å, Zheng H (2020). Amino acid-functionalized two-dimensional hollow cobalt sulfide nanoleaves for the highly selective enrichment of N-linked glycopeptides. Anal Chem.

[24] Balachandran S, Jeeva Jothi K, Selvakumar K, Bhat DK, Sathiyanarayanan K, Swaminathan M (2020). Solar active ZnO-Eu2O3 for energy and environmental applications. Mater Chem Phys.

[25] Balachandran S, Karthikeyan R, Jothi KJ (2022). Fabrication of flower-like bismuth vanadate hierarchical spheres for an improved supercapacitor efficiency. Mater Adv.

[26] Jothi KJ, Kumar RD, Hasan I (2024). One-pot synthesis of morinda pubescens fruit-like structure of Bi@BiVO4 by a simple hydrothermal route: high performance and long-term stability for supercapacitor applications. J Energy Storage.

